# The Function of the Alula in Avian Flight

**DOI:** 10.1038/srep09914

**Published:** 2015-05-07

**Authors:** Sang-im Lee, Jooha Kim, Hyungmin Park, Piotr G. Jabłoński, Haecheon Choi

**Affiliations:** 1 Institute of Advanced Machines and Design, Seoul National University, Seoul, Korea; 2 School of Biological Sciences, Seoul National University, Seoul, Korea; 3 Department of Mechanical & Aerospace Engineering, Seoul National University, Seoul, Korea; 4 Museum of Zoology, Polish Academy of Sciences, Warsaw, Poland

## Abstract

The alula is a small structure located at the joint between the hand-wing and arm-wing of birds and is known to be used in slow flight with high angles of attack such as landing. It is assumed to function similarly to a leading-edge slat that increases lift and delays stall. However, in spite of its universal presence in flying birds and the wide acceptance of stall delay as its main function, how the alula delays the stall and aids the flight of birds remains unclear. Here, we investigated the function of alula on the aerodynamic performance of avian wings based on data from flight tasks and wind-tunnel experiments. With the alula, the birds performed steeper descending flights with greater changes in body orientation. Force measurements revealed that the alula increases the lift and often delays the stall. Digital particle image velocimetry showed that these effects are caused by the streamwise vortex, formed at the tip of the alula, that induces strong downwash and suppresses the flow separation over the wing surface. This is the first experimental evidence that the alula functions as a vortex generator that increases the lift force and enhances manoeuvrability in flights at high angles of attack.

The alula is a small structure that is composed of a digit bone and two to six feathers^1^. Its presence is universal in extant flying birds and can also be found in the fossils of several early ancestors of birds^2^,^3^,^4^. It has long been assumed that the alula functions in a similar manner to that of the extended leading-edge slat in an aircraft, which increases the lift force at high angles of attack with delaying the stall^6^. Thus, it has been widely accepted that the alula aids slow flights or flights at high attack angles such as landing^1^,^5^,^7^. Among the previous studies that have attempted to reveal the aerodynamic mechanism of alula functioning as a leading-edge slat^7^,^8^,^9^,^10^, some found the lift enhancing effect of the alula^7^,^9^,^10^ (in one study, one out of four specimens had measurable lift enhancement^10^). Particularly, the classic paper by Nachtigall and Kempf^7^ rigorously conducted force measurements on the dried wings of three avian species and described how birds used the alula in landing. However, not all the previous studies affirmed the stall-delaying effect of the alula and the results of those studies were not conclusive and failed to provide a generalized view on the function of alula in actual flights of birds. Also, the alula is a three-dimensional structure, so its function may not be similar to the stall-delaying effect of two-dimensional leading edge slat^11^. Recently, two possible three-dimensional effects of the alula were hypothesized^5^,^6^: (i) the induction of leading edge vortex over the hand-wing (distal part of the wing) and (ii) the prevention of the spanwise thickening of the boundary layer (which otherwise would encourage flow separation) by inhibiting the spanwise flow with small-scale vortices created by the alula (i.e., similar to the wing fence and vortilon used in backward-swept wing aircraft). However, no experiments have been conducted to evaluate these hypotheses. In the present study, we conducted field observations and force and velocity measurements to evaluate the hypotheses regarding the three-dimensional effects of the alula in avian flight.

In order to describe how the alula affects the flight of birds, we analysed the kinematics of free descending flights of four juvenile captive Eurasian magpies (*Pica pica*). From a 170 cm-high perch, the birds landed on the ground after three to four wingbeats. They performed steep and fast descents during the first wingbeat, and leveled their bodies during or at the end of the second wingbeat. During the second wingbeat, the speed was greatly reduced and the deflection of secondary coverts was visible which indicates flow separation over the suction surface of the wing^2^. When the alula was present, the duration of total descending flight was shorter (GLMM, *F*_1,20_ = 9.76, *P* = 0.005; Fig. 1 (a)). During the second wingbeat, the inclined angle of flight path (angle of descent) was higher (*t*_67_ = −2.14, *P* = 0.036; Fig. 1 (b)), and the change in the body orientation was greater (*t*_68_ = −2.3, *P* = 0.025; Fig. 1 (c)) when the alula was present. The sinking speed (the vertical distance travelled during a wingbeat divided by the duration of a wingbeat) was not influenced by the presence of the alula (Fig. 1 (d); detailed statistical results are given in Table S1). Our results suggest that the use of alula should enable the bird to perform steeper descending flights with greater changes in body orientation while it does not decelerate the sinking speed. On the other hand, in our study, the deflection of the alula was visible as early as at the first downstroke where the airspeed around the wing is approximately 2.0 ms^−1^ (Fig. 1 (d)) which implies that the use of the alula may be actively controlled by the birds. This is in contrast to an earlier observation of the passive deflection of the alula against the wind speed that exceeds 12 ms^−1^ in a live pigeon^8^. Currently it is not clear whether the alula deflection is actively or passively controlled by birds. More rigorous studies on how the alula is deflected depending on the flight speed and whether it is actively controlled are needed.

We performed a series of wind-tunnel experiments to examine the aerodynamic performances of bird wings with and without an alula. We measured the lift and drag forces at free-stream velocities of *U*_0_ = 3 − 15 ms^−1^ (corresponding Reynolds numbers, based on the mean wing chord length, are *Re_c_* ~ 0.2 − 1.3 × 10^5^) for three fixed wings (UJ07, UJ13, and KS20), and at *U*_0_ = 3 ms^−1^ for one fixed wing (UJ30) of adult male magpies (detailed information is given in Table S2 and Fig. S1 in the Supplementary Materials). Polar plots of lift-area (*C_L_A,* see Methods for definition) versus drag-area (*C_D_A*) showed that the alula has a beneficial effect on the lift-area at high angles of attack when the drag-area is large (Fig. 2). That is, when the lift force increased rapidly but the drag force varied slightly (i.e. at low angles of attack), the alula had a small effect on the polar curve. However, when the lift force was maintained at a nearly constant value but the drag force increased greatly (i.e. at high angles of attack), the lift force was clearly elevated by the presence of the alula. Although the magnitude of lift enhancement differed among the wings, the general pattern was consistent. While the drag force changed little owing to the presence of the alula at any range of angles of attack (Figs. S2–S4; *P* > 0.85 in all four wings tested), the lift force increased with the alula at higher angles of attack (Figs. S2–S4; see Methods for the range of attack angles used for statistical comparison; *F*_1,108_ = 38.57, *P* < 0.0001 in UJ07; *F*_1,94_ = 27.10, *P* < 0.0001 in UJ13; *F*_1,9_ = 26.96, *P* = 0.0006 in UJ30; *F*_1,94_ = 2.91, *P* = 0.091 in KS20). Although the lift enhancement was marginally non-significant in KS20, the trend remained the same and the opposite pattern was never observed. Especially, in UJ13, the variations in the lift coefficients showed that the alula increased the lift force by 1.3–12.7% (6.12 ± 2.64%, mean±SD) and delayed the stall by 5°–10° (Fig. S3). Combining the results from the present force measurement and field observations, we propose that the alula enables birds to be more manoeuvrable (i.e., high rate of change in the body orientation without degradation of sinking speed) by generating larger lift forces at high angles of attack.

We measured the velocity field using DPIV to examine the modification of the flow structures around the bird wing (UJ30) at the attack angle of 24° by the presence of the alula (see Fig. S6 for detailed setup), at which the lift force was increased by the amount of 6.68% with the alula. Although using fixed wings may underestimate the possible unsteady effect^12^, earlier studies on fixed bird wings successfully examined the flow structure and aerodynamics of the wings^13^,^14^. Fig. 3 (a) shows the contours of the time-averaged streamwise velocity on *x-y* planes at five spanwise locations on the wing. No discernible change in the streamwise velocity by the presence of the alula was found near the root of the alula (location *z_1_*). However, with the alula, the flow separation occurred farther downstream and thus the flow was attached more to the upper surface of the wing at the spanwise locations of mid span (*z_2_*) and the tip of the alula (*z_3_*). The shear layer near the leading edge was thinner with the alula (inset of Figure 3 (a)). Even in the regions away from the tip of the alula (*z_4_* and *z_5_*), flow was attached to the upper surface and the separated region in the wake became smaller. Although the flow passing through the gap between the alula and the leading edge of the wing was not clearly captured by the current velocity measurement (owing to the blockage caused by three-dimensional structure of the wing), it can be deduced that the flow around the alula enables the flow over the wing surface to resist the adverse pressure gradient and thus the flow separation is delayed farther downstream (see also Fig. 3 (b) below).

As for the mechanism responsible for the separation delay by the alula, we suggest a vortex formed at the tip of alula feathers. Shown in Fig. 3 (b) are the contours of the instantaneous streamwise vorticity on *y*-*z* planes at different streamwise locations on the wing. It is clearly visible that a strong streamwise vortex rotating in the counter-clockwise direction was formed at the tip of the alula and travelled downstream in the streamwise direction. This tip vortex (first appeared at *x_2_*) induced vortices rotating in the clockwise direction, which are visible at *x_3_* and *x_4_*. As the flow went downstream, the alula-tip vortex moved upward from the upper wing surface. The alula-tip vortex created a strong downwash near the tip of alula (see inset of Fig. 3 (b)), which would add significant streamwise momentum to the flow near the wing surface. This effect explains the decrease in the shear layer thickness and thus the suppression of flow separation over the wing surface when the alula was present (Fig. 3 (a)). This effect of the alula is similar to that of a tilted vortex generator (VG) in that both generate a streamwise vortex which enhances the momentum transport from the free-stream flow to the low-speed flow near the wing surface^15^,^16^,^17^,^18^,^19^. Note that the streamwise vortex rotating in the counter-clockwise direction by the alula generated additional spanwise flow from the wing root to the tip, directing near-surface streamlines towards the wing tip. Thus, the suppression of flow separation is more pronounced at the region near and outside the tip of the alula (see Fig. 3 (a); at *z_2_* to *z_5_*) than at the root of the alula (Fig. 3 (a); at *z_1_*). This indicates that the alula is more responsible for the change in flow structure on the hand-wing rather than that on the arm-wing. The suggested mechanism of the separation delay by the alula-tip vortex is illustrated in the artist’s impression of the flow around the wings of a landing bird in Fig. 3 (c).

When the wings are at high angles of attack, the vertical distance between the alula-tip vortex and the upper wing surface becomes larger. Considering that the viscous dissipation near the wing surface may cause a vortex to lose its circulation^20^, the alula-tip vortex keeping a certain distance from the wing surface should prevent the wing from losing its circulation too quickly. Also, the chord line of the alula is negatively deflected from that of the main wing (e.g., the angle between two chord lines is −29° in UJ30). Therefore, unless the angle of attack is high enough, the vortex generated from the alula tip may not work properly in inducing high momentum towards the suction surface of the wing. This may explain why the alula is effective in suppressing flow separation at high angles of attack. Our results provide the first experimental evidence that the alula aids birds’ flight owing to a streamwise vortex generated at the tip of the alula. Although lift enhancing effect of the alula was also found in some of previous studies^7^,^9^, our DPIV results provide novel explanations on how the lift enhancement is achieved. The alula-tip vortex is the key mechanism for lift enhancement and stall delay at high angles of attack which endows birds with greater flight manoeuvrability. Based on these results, we suggest that the alula may be used in any flight that involves high angles of attack (such as turning flights^7^), and that more detailed studies on the contexts of alula usage are warranted.

## Methods

### Flight tasks

In order to compare performance in descending flight tasks with regard to the presence or absence of an alula (i.e. before and during moulting alula feathers), we analysed free descending flights of four juvenile captive Eurasian magpies (*Pica pica*) filmed in an aviary with a high speed camera at 125 fps (Troubleshooter 1000 ME, Fastec Imaging). The birds were trained for two weeks prior to the tasks to fly down to obtain food items located just beneath them on the ground from a 170 cm-high perch. In most cases, the birds descended steeply (at an inclined angle larger than 50° against the horizontal line) and only these data were analysed. For each test, individuals were filmed twice within a 5-day interval, during which alula feathers were fully grown whereas the moulting process of other parts of the body remained unchanged. This is consistent with what is known with the moulting process of postjuvenile molt^21^. Hence, the first test for each bird comprised the ‘without the alula’ condition and the second comprised the ‘with the alula’ condition. It is unlikely that the first test affected the second one within a five-day interval, and we do not see how other factors apart from the recovery of the alula can cause a systematic change of the flight performance of the birds. By analysing the video clips, we obtained the duration, angle of descent (angle between the flight path and the horizontal line), change in body orientation and sinking speed (vertical distance travelled during a wingbeat divided by the duration) during each wingbeat and total flight, respectively, before and after the alula feathers were recovered. We carefully chose the video clips where we could measure the angle of descent with confidence and discarded the video clips that did not allow the analysis of angle of descent (such as the cases when the descent did not happen in the plane perpendicular to the camera lens axis, which was evaluated by an observer located in this plane). Also, we did not have exact measurements of the heading angle or body orientation. Thus, we estimated the body orientation at the end of each wingbeat by comparing the view of the image with that of known body orientations of a stuffed magpie and categorized the changes in the body orientation into four groups. If the bird did not show any discernible change in body orientation during the flight, the body orientation was coded as “1”; if the change in body orientation was estimated to be higher than 0° and lower than 45°, it was coded as “2”; if it was between 45° and 90°, it was coded as “3”; if it was higher than 90°, it was coded as “4”. We analysed a total of 28 descending flights (18 and 10 flights with and without alula, respectively). The effect of alula presence was analysed with a general linear mixed model (GLMM) where the bird identity was treated as a random factor. We conducted planned comparisons for comparing the duration, angle of descent, change in body orientation and sinking speed between alula-present and alula-absent conditions. Statistical analysis was conducted with SAS version 9.3 (SAS Institute, Cary). All experiments were performed in accordance with relevant guidelines and regulations of Seoul National University. All experimental protocol were approved by Institutional Animal Care and Use Committee of Seoul National University (No. SNU 130621-6).

### Force measurements

We used four fixed wings of adult male magpies (UJ07, UJ13, UJ30 and KS20) for force measurements. The wings were prepared by severing the shoulder joint (between humerus and scapula) and were dried in a landing posture with the alula deflected (detailed information is given in Table S2 and Fig. S1 in the Supplementary Materials). The angle between the alula and distal primaries and that between the alula and leading edge of the wing were determined based on high speed video clips of landing of another captive magpie that was not used in the flight task. The angle of attack was defined as the angle between the direction of free-stream and the line that connects the leading edge to the trailing edge (i.e. tip of the secondary feathers) from the view at the wing root with no wind condition. Lift and drag forces with and without (i.e. severed) alula feathers were measured with a load cell (CAS BCL-1L). The resolution of the load cell was 0.001 N with a maximum capacity of 10 N. For UJ07, UJ13 and KS20, the free-stream velocities were 3, 5, 7, 9, 11, 13 and 15 ms^−1^ with varying angles of attack from 0° to 60° (UJ07, KS20) or to 70° (UJ13) with increments of 5°. UJ30 was tested only at the free-stream velocity of 3 ms^−1^ with angles of attack from 0° to 60° with increments of 5°. These free-stream velocities corresponded to the Reynolds numbers based on the mean chord length of *Re_c_* = *U_o_c*/*ν* = 0.2 − 1.3 × 10^5^, where *U_o_* is the free-stream velocity, *c* is the mean chord length, and ν is the kinematic viscosity of air. The signals from the force sensor were digitized by an A/D converter (PXI-6259, National Instruments Co., Austin, TX, USA) and sampled for 300 s at a rate of 10 kHz to obtain a fully converged mean value. The voltage outputs from the sensor were calibrated using the calibration matrix supplied with the sensor. Forces were measured three to five times for each sample and the average values are reported here. The repeatability errors of force measurements were within ± 1.7%. The variations in the force profiles were shown in Fig. S5. The drag and lift of the strut were measured separately and used for the correction of those measured with each wing. As the planform of fixed wing changed with the angle of attack, we used the lift-area and the drag-area in describing the flight performance of the wing. The lift-area (*C_L_A*) and drag-area (*C_D_A*) coefficients were calculated as *C_D_A* = *D*/(0.5*ρU_o_*^2^) and *C_L_A* = *L*/(0.5*ρU_o_*^2^), respectively, where *D* is the drag force, *L* is the lift force, *ρ* is the air density, and *A* is the planform area of the wing. Two wings (UJ07, UJ13) were tested in an open-type wind tunnel with the test section size of 0.6 × 0.6 m^2^. The other two fixed wings (KS20, UJ30) were tested in a closed-type wind tunnel with the test section size of 0.9 × 0.9 m^2^. The corresponding blockage ratios when the angle of attack was 20° were below 4% and 2% for the open-type and closed-type wind tunnels, respectively.

Forces measured with and without the alula were compared using a general linear mixed model (SAS ver 9.3, SAS Institute, Cary). Data from wings were treated separately, as they differed in the range of angles of attack and in the magnitude of lift enhancement by the alula. The angles of attack where the alula increased the lift forces were above 25° in UJ07, above 40° in UJ13, between 20° and 40° in UJ30, and above 25° in KS20.

### Digital particle image velocimetry (DPIV)

With UJ30, we used DPIV to obtain the velocity and vorticity around the wing with and without the alula. The measurements were performed at the free-stream velocity of 3 ms^−1^ and at an angle of attack of 24° which was the condition for the maximum lift-area of the wing (see Fig. S4). The schematic diagram for DPIV is shown in Fig. S6. A closed-type wind tunnel with the test section size of 0.9 × 0.9 m^2^ was used. The end plate of 14 mm thickness, extending 200 mm upstream of the leading edge with a rounded edge, was installed to mitigate end-wall effects. The angle of attack of the wing was set using a rotating stage with an angular resolution of 0.03°. The DPIV system consisted of an Nd:Yag laser (Solo 120, New Wave Research, USA), a timing hub (Integrated Design Tools), a fog generator (F2010, Safex), and a CCD camera mounted with an optical lens (Nikon). The fog generator produced liquid droplets of approximately 1 μm in diameter, which were introduced into the wind tunnel. Five planes parallel to the *x*-*y* plane and four planes parallel to the *y*-*z* plane were illuminated from one side of the wind tunnel with a thin laser light of 3 mm thickness using laser optics. Iterative cross-correlation analysis was performed with an initial window size of 64 × 64 pixels and with 32 × 32 final interrogation windows. The interrogation window was overlapped by 75%, leading to spatial resolutions of about 1.16 mm (0.0074 *c*) for planes parallel to the *x*-*y* plane and about 1.07 mm (0.0068 *c*) for planes parallel to the *y*-*z* plane, where *c* is the mean chord length. To obtain the time-averaged flow field, 2,000 pairs of images were collected and processed.

## Author Contributions

S.I.L. and P.G.J. conducted flight tasks and statistical analyses; S.I.L., J.K. and H.P. conducted force measurements and DPIV; S.I.L., J.K., H.P., P.G.J. and H.C. wrote the paper.

## Supplementary Material

Supplementary Information Supplementary information

## Figures and Tables

**Figure 1:**
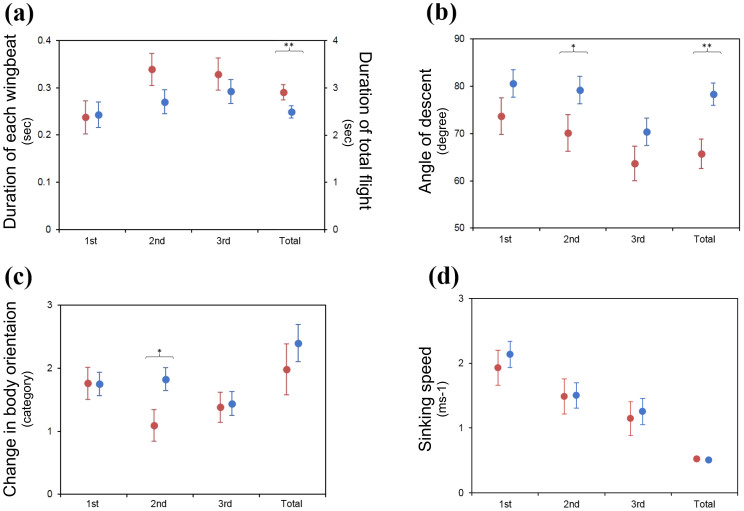
Comparison of descending flights of four Eurasian magpies during alula molting (‘without the alula’, red circles) and after recovery of alula(‘with the alula’, blue circles). Data are represented for each wingbeat separately and for total flight. Error bars denote the standard errors. Significance levels of statistical comparisons were denoted as “*” for 0.01 <*P* <0.05 and “**” for 0.001 <*P* <0.01.

**Figure 2:**
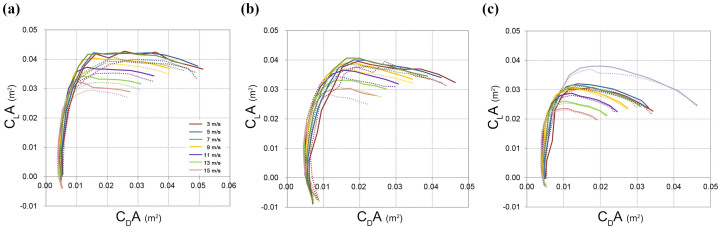
Force measurements: glide polar based on the lift-area (*C_L_A*) and the drag-area (*C_D_A*). Results from (a) UJ07, (b) UJ13, (c) KS20 and UJ30(curves in lavender are for UJ30 at *U*_0_ = 3 ms^−1^), with the alula (solid lines) and without the alula (dotted lines). Different colors represent different free-stream velocities.

**Figure 3:**
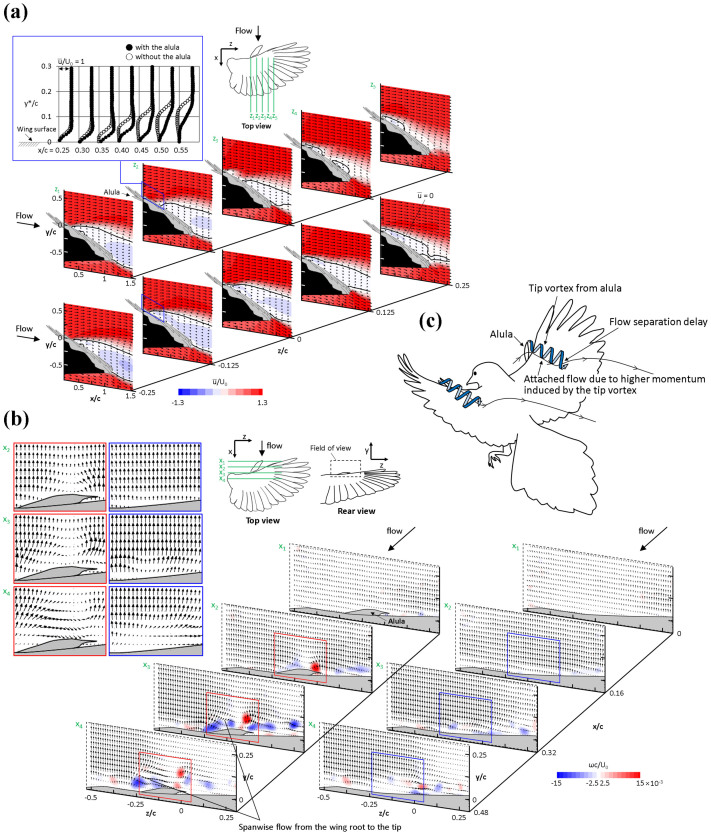
Aerodynamic mechanism for the lift enhancing effect of alula. (a) Contours of the time-averaged streamwise velocity (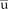) at five spanwise locations on the wing (UJ30): with the alula (upper panel) and without the alula (lower panel). The angle of attack was 24°. Axis values were normalized by the mean chord length (*c*), and the velocity values were normalized with the free-stream velocity (*U*_0_) which was 3 ms^−1^. Arrows denote the velocity vectors. The inset shows the profiles of the mean streamwise velocity above the wing surface at *x/c* = 0.25 − 0.55 for the location z_2_ with (solid circle) and without the alula (open circle). The origin (*x* = 0, *y* = 0, *z* = 0) was located at the tip of the alula when unfolded and y* in the inset denotes the distance from the wing surface. Solid black lines in the contours denote the locations where the mean streamwise velocity is zero. With the alula, except for z_1_ (near the root of the alula, leftmost figures), the flow separation starts to occur farther downstream and thus the flow is attached more to the upper surface of the wing. This effect of the alula on the streamwise velocity is still observed at the regions away from the alula tip (*z*_4_, *z*_5_). Black-colored areas in these figures represent the regions that were shadowed by the wing. (b) Contours of the instantaneous streamwise vorticity (*ω*) at four streamwise locations on the wing (UJ30): with the alula (left panel) and without the alula (right panel). A vortex that is created at the tip of the alula and rotates in the counter-clockwise direction is clearly visible. The inset figures on the left top corner represent the mean velocity vector fields around the location of the alula. Vorticity values were normalized with the chord length and the free-stream velocity (*U*_0_) which was 3 ms^−1^. Arrows denote the velocity vectors. (c) Artist’s impression on the effect of the alula-tip vortex on the flow separation. The figure was drawn by the authors.
