# Supplementary material for: The Function of the Alula in Avian Flight
Source: Sci Rep. 2015 May 7;5:9914. doi: 10.1038/srep09914 (PMC4423427; doi:10.1038/srep09914)
Supplement: Supplementary Information [file srep09914-s1.pdf]

Supplementary information of the paper titled “The Function of the Alula in Avian Flight”

by Sang-im Lee, Jooha Kim, Hyungmin Park, Piotr G. Jabłoński and Haecheon Choi

Table S1. Detailed statistical results on the comparisons of the flights of four juvenile magpies with respect to the presence of the alula.
Table S2. Morphometric characteristics of bird wing samples used in this study.
Figure S1. Experimental setup for the force measurements and definitions of sample characteristics.
Figure S2. Variations of the drag-area, lift-area, and lift-to-drag ratio with the angle of attack, measured for UJ07.
Figure S3. Variations of the drag-area, lift-area, and lift-to-drag ratio with the angle of attack, measured for UJ13.
Figure S4. Variations of the drag-area, lift-area, and lift-to-drag ratio with the angle of attack, measured for KS20 and UJ30.
Figure S5. Raw data of the force measurements on UJ07.
Figure S6. Experimental setup for DPIV.

Table S1. Detailed statistical results on the comparisons of the flights of four juvenile magpies with respect to the presence of the alula. The table shows t statistics for the planned comparisons of the flight performed during each wingbeat, and F statistics for the comparisons of total flight.

	Planned comparisons			Total flight
	1st wingbeat	2nd wingbeat	3rd wingbeat	
Duration	t_{71} = -0.39, P = 0.696	t_{71} =1.64, P = 0.105	t_{71} = 0.73, P = 0.465	$F_{1,20}$ = 9.76, P = 0.005
Angle of descent	t_{67} = -1.41, P = 0.162	t_{67} = -2.14, P = 0.036	t_{67} = -1.42, P = 0.159	$F_{1,20}$ = 10.33, P = 0.004
Body orientation	t_{68} = 0.09, P = 0.930	t_{68} = -2.3, P = 0.025	t_{68} = -0.12, P = 0.902	$F_{1,20}$ = 0.64, P= 0.435
Sinking speed	t_{68} = -0.69, P = 0.494	t_{68} = -0.09, P = 0.926	t_{68} = -0.41, P = 0.680	$F_{1,20}$ = 0.01, P = 0.905

Table S2. Morphometric characteristics of bird wing samples used in this study.

Sample name	UJ07	UJ13	KS20	UJ30
Body mass (g)	250.9	245.0	256.3	241.4
Wing area (single wing, projected area, cm^2)	362.0	331.1	353.6	440.0
Wing loading (single wing, kg m^{-2})	6.93	7.40	7.25	5.49
Wing span (single wing, cm)	24.1	22.4	27.3	28.0
Wing chord (average, cm)	12.2	11.9	13.0	15.7
Wing chord (maximum, cm)	13.3	13.0	19.5	20.0
Wing aspect ratio	1.60	1.52	2.10	1.78
Alula span (cm)	5.9	5.8	5.2	6.0
Alula chord (cm)	1.3	1.1	1.0	1.1
Alula chord (in relation to maximum wing chord)	9.8%	8.5%	5.1%	5.5%
Alula position (from wing root, in relation to the wing span)	25.9%	30.0%	25.0%	29.6
Angle between the alula and distal primary (degree)	22	25	23	25
Angle between the alula and leading edge (degree)	136	135	136	135

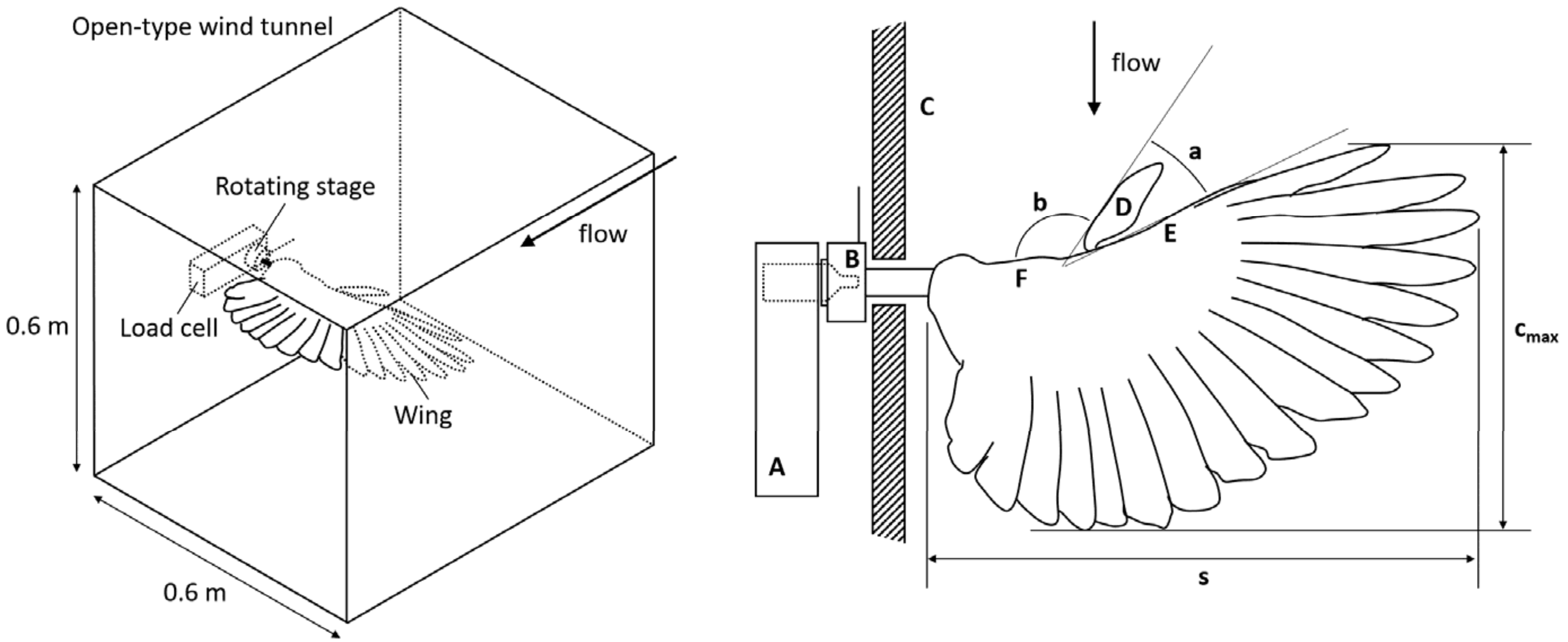

Figure S1. Experimental setup for the force measurements and definitions of sample characteristics: A, load cell; B, rotating stage with an angle indicator; C, wind-tunnel chamber; D, alula; E, distal primary; F, leading edge of the wing; *s*, wing span; c_{max}, maximum wing chord; *a*, angle between the alula and distal primary (planform); *b*, angle between the alula and leading edge of the wing (planform).

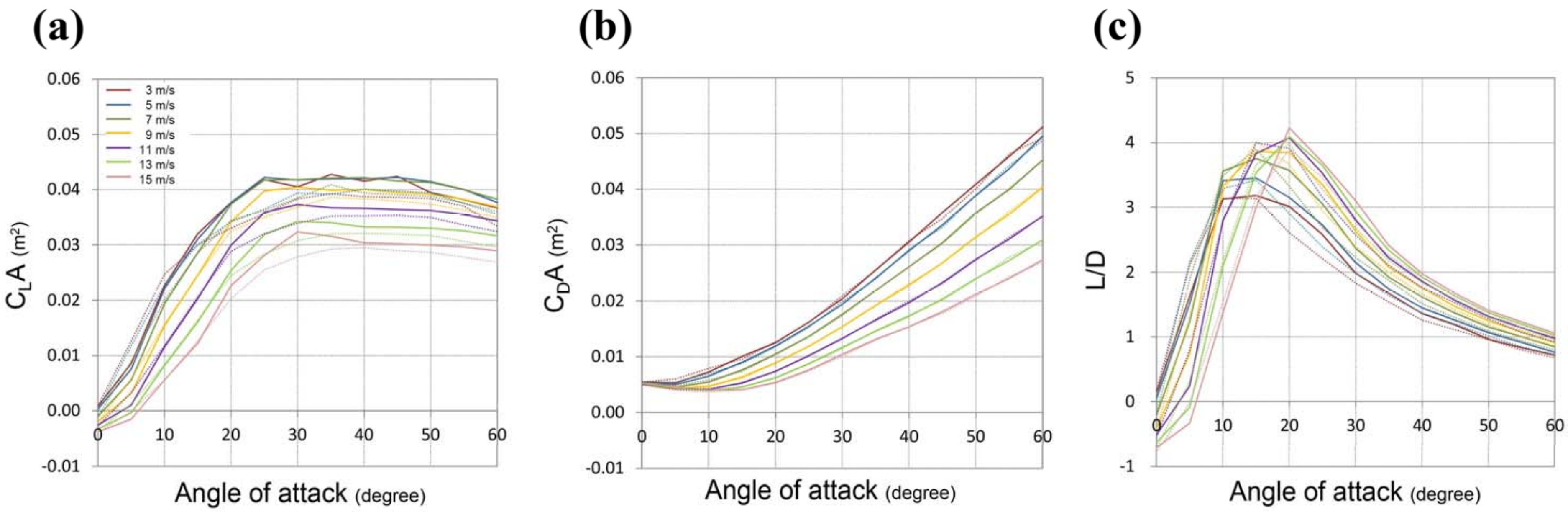

Figure S2. Variations of the drag-area, lift-area, and lift-to-drag ratio with the angle of attack, measured for UJ07: solid lines: with the alula; dotted lines: without the alula.

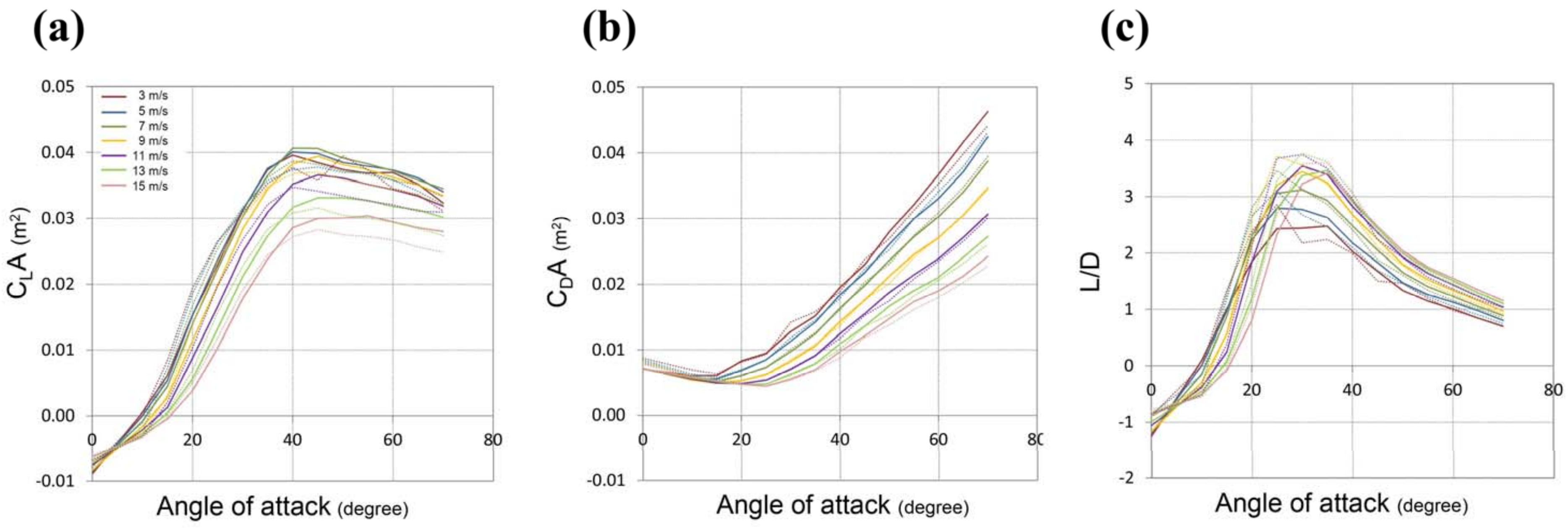

Figure S3. Variations of the drag-area, lift-area, and lift-to-drag ratio with the angle of attack, measured for UJ13: solid lines: with the alula; dotted lines: without the alula.

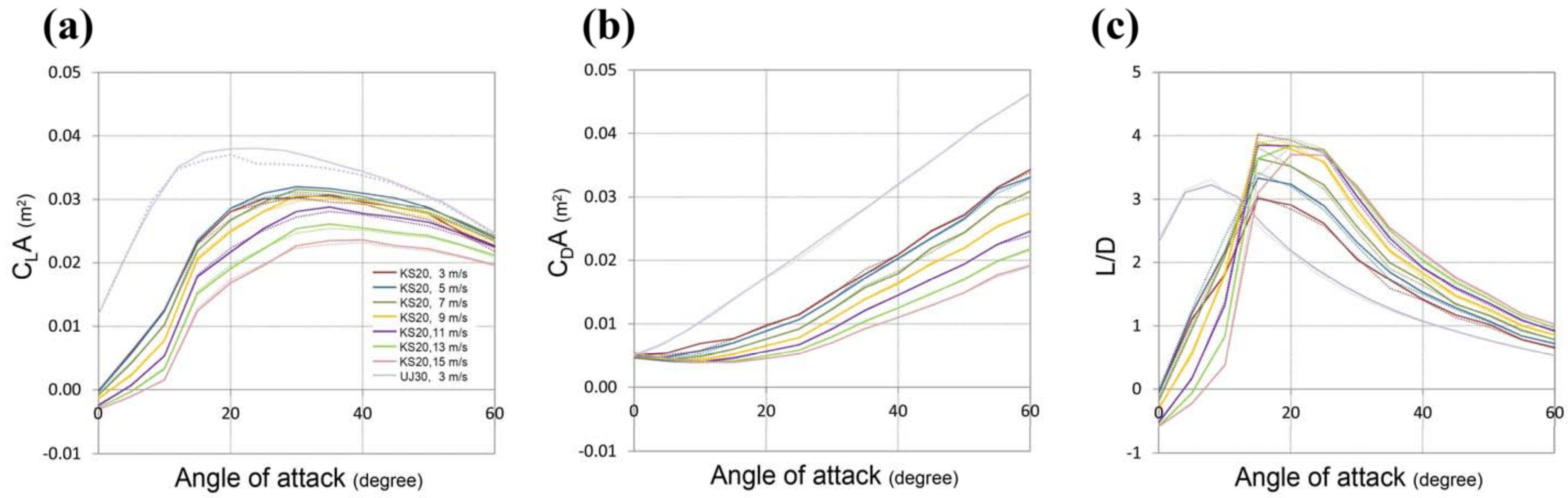

Figure S4. Variations of the drag-area, lift-area, and lift-to-drag ratio with the angle of attack, measured for KS20 and UJ30: solid lines: with the alula; dotted lines: without the alula. Curves in lavender are for UJ30 at U_0 =3 ms^{-1}.

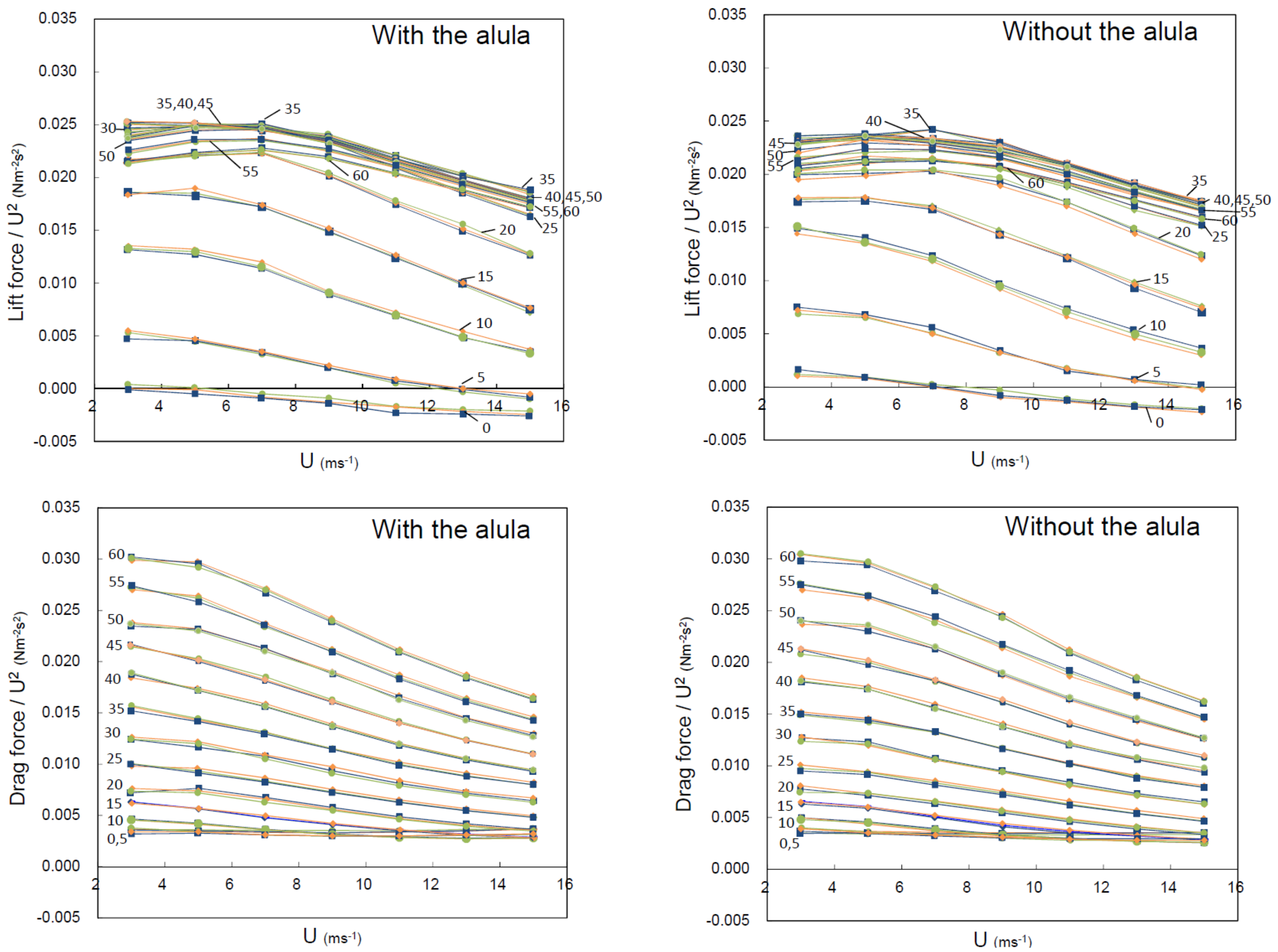

Figure S5. Raw data of the force measurements on UJ07. Different symbols overlapping with one another represent the force values measured at the same angle of attack repeatedly. Numbers in the graphs denote the angles of attack.

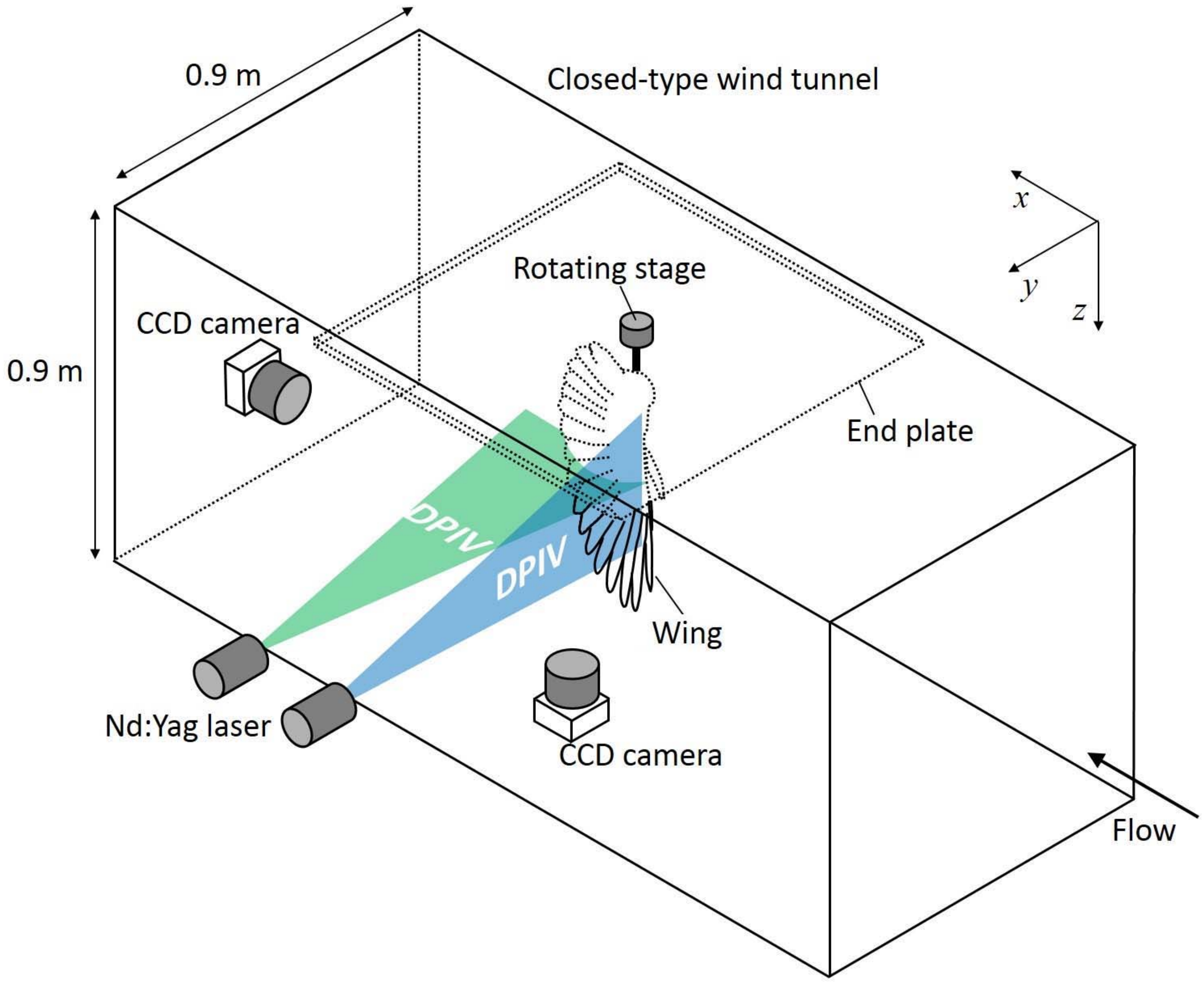

Figure S6. Experimental setup for DPIV.
